# Effect of Dilution on Microstructure and Phase Transformation of AlCrFeMnNi High-Entropy Alloy by Resonant Ultrasonic Vibration-Assisted Laser Cladding

**DOI:** 10.3390/ma18030695

**Published:** 2025-02-05

**Authors:** Aziz Ul Hassan Mohsan, Mina Zhang, Menggang Zhai, Yishen Wang, Mudaser Ullah, Xuedao Shu, Su Zhao

**Affiliations:** 1 Faculty of Mechanical Engineering and Mechanics, Ningbo University, Ningbo 315211, China; hassan@nimte.ac.cn; 2 Ningbo Institute of Materials Technology and Engineering, Chinese Academy of Sciences, Ningbo 315201, China; zhaimgmail@nimte.ac.cn (M.Z.); wangyishen@nimte.ac.cn (Y.W.); 3 Department of Mechanical Engineering, Technological University Dublin, D01 K822 Dublin, Ireland; mudaser.ullah@tudublin.ie

**Keywords:** ultrasonic vibration-assisted laser cladding (UVALC), dilution, microstructure, tribology, mechanical properties

## Abstract

The present study effectively produced a high-entropy alloy (HEA) coating of AlCrFeMnNi on AISI 304L steel using resonant ultrasonic vibration-assisted laser cladding (R-UVALC). An investigation was conducted to examine the impact of dilution rate on the phase composition, microstructure, and mechanical and tribological properties of AlCrFeMnNi coatings. The coating, which was created utilizing the appropriate dilution rate, was thoroughly characterized using EDS mapping and TEM investigation. The results suggest that a higher dilution rate causes a change in the AlCrFeMnNi coating, transforming it from a single solid solution phase (BCC) into a two-phase solid solution containing both FCC and BCC phases. The analysis conducted using transmission electron microscopy (TEM) reveals that the AlCrFeMnNi coating, when diluted at an optimal rate of around 37%, is predominantly composed of a disordered body-centered cubic (BCC) phase and an ordered BCC (B2) phase featuring a spinodal decomposition structure. The AlCrFeMnNi coating has an average microhardness of approximately 540 HV, which is over 2.5 times higher than the microhardness of the substrate. Additionally, it was also established that the dilution rate has an impact on the occurrence of phases, which subsequently affects the mechanical and antifrictional properties of the coating. The integration of ultrasonic vibration in laser cladding enhances quality and improves mechanical and tribological properties, thereby reducing material costs and promoting an environmentally friendly process when compared to conventional cladding.

## 1. Introduction

Metallurgical research aims to improve material properties in order to meet the requirements of high-performance industries. Conventional metal alloys, which are created by combining a main element with smaller elements, require meticulous control to prevent them becoming brittle and forming unwanted solid particles. Phase diagrams are utilized for the purpose of controlling the proportions of elements, resulting in the development of high-entropy alloys (HEAs), as discovered by Cantor and Yeh [1,2]. It was discovered that when at least five elements are combined in equal amounts, it diverges from conventional techniques. This leads to the formation of stable solid solutions and prevents the creation of intermetallic compounds. High-entropy alloys (HEAs) possess remarkable strength and exhibit resistance to thermal softening, wear, and corrosion. These coatings, such as AlCoCrFeNiNbx [3], FeCoNiCrMnTi [4], AlxCoCrFeNi [5], Fe_50_Mn_30_Co_10_Cr_10_ [6], FeCrFeNiNbx [7], and FeNiCoCrMox [8], are commonly employed in laser cladding on 304 steel. FeCoCrMnNi alloys with equiatomic composition, especially when aluminum is added and cobalt is removed, exhibit a strong body-centered cubic (BCC) phase, enhancing their mechanical and wear-resistance properties [9]. However, the process of laser cladding AlCrFeMnNi is challenging because of its susceptibility to brittleness and cracking. These concerns can be mitigated by utilizing ultrasonic vibration, which improves performance by leveraging thermal and acoustic effects during the solidification process. Ultrasonic vibration enhances laser welding and cladding processes by minimizing cracks, refining grain structure, and removing pores. The profiles of metal droplets are altered by the combined effects of gravity, surface tension, and ultrasonic vibration effect, resulting in changes in energy distribution and modifications in their trajectory. Droplets experience elastic deformation with minimum oscillation, but more oscillation can hinder their separation from the substrate. The droplets are held in position by the forces of gravity, surface tension, and the inertial forces generated by ultrasonic vibration [10].

Shon et al. [11] laser-cladded AlFeCrCoNi high-entropy alloy onto aluminum substrate using laser cladding with a pre-coating step, managing dilution by employing double-layered coatings and increasing energy input, but they encountered issues with cracking. Mohsan et al. [12] experimented with AlCrFeMnNi HEA using a resonant ultrasonic vibration-assisted cladding setup, achieving exceptional mechanical and tribological properties. Zhu et al. [13] investigated the Inconel 718 coating using ultrasonic vibration-assisted laser cladding (UVALC), and found that this technique influences the nucleation rate and undercooling degree, resulting in finer grain sizes compared to those obtained by conventional methods. Zhang et al. [14] explored ceramic particle-reinforced Fe-based composite coatings under UVALC, noting enhanced coating profiles but increased dilution. While multiple studies have shown extraordinary results with UVALC, they lack uniformity in terms of ultrasonic vibration application, affecting dilution rates and overall product quality. Further research is needed to assess dilution effects by utilizing the resonant ultrasonic vibration-assisted laser cladding (R-UVALC) technique on phase, microhardness, and mechanical properties.

Precise control of dilution is essential in laser cladding to produce clad coatings with superior performance. Attaining a complete metallurgical connection and ideal degrees of dilution are crucial, as dilution has a major impact on the mechanical and corrosion-resistant characteristics of the coating [15,16]. Excessive energy that causes the substrate material to melt might lead to an over-dilution of the coating composition, which, in turn, leads to a deterioration in its qualities. On the other hand, if there is not enough heat energy, it can stop the fusion bond from forming, which can result in weak adhesion and the possibility of cladding spalling during use [17]. The inclusion of ultrasonic vibrations during the laser cladding results in an amplified dilution effect due to the presence of acoustic streaming and acoustic cavitation, which increases the absorption of laser energy to its highest potential level. The introduction of the substrate into the coating leads to alterations in microstructure and phase, thereby impacting the mechanical and tribological characteristics’ performance. This work investigates the impact of dilution on the microstructure phase transformation and mechanical and tribological properties through the application of resonant ultrasonic vibration-assisted laser cladding (R-UVALC) on a high-entropy alloy of AlCrFeMnNi.

## 2. Experimental Procedures

**Figure 1:**
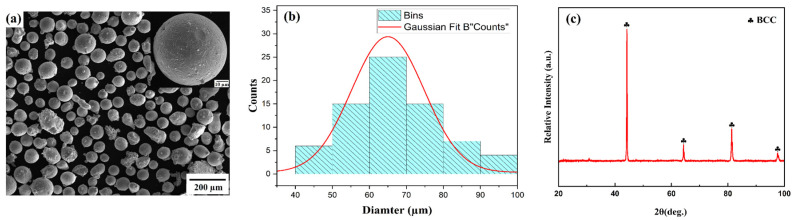
(**a**) SEM morphology of the AlCrFeMnNi high-entropy alloy powder particles, (**b**) particle size distribution of the AlCrFeMnNi, and (**c**) XRD of the high-entropy alloy powder.

**Figure 2:**
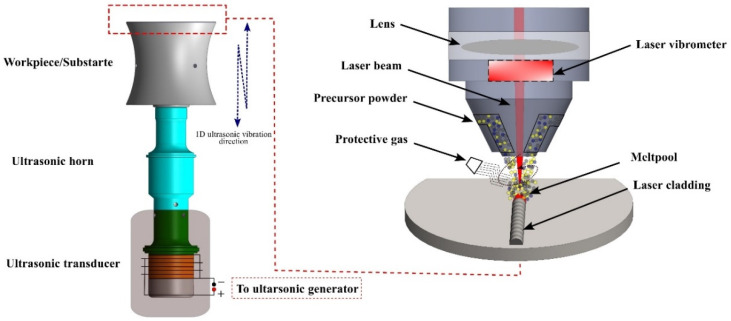
The experimental configuration of the resonant ultrasonic vibration-assisted laser cladding (R-UVALC).

The HEA powders utilized in this investigation were produced by the process of gas atomization. Figure 1a–c display the scanning electron microscope (SEM), particle size distribution, and X-ray diffraction (XRD) patterns of the pre-alloyed powders, respectively. The XRD pattern in Figure 1c reveals that the main phase present is a body-centered cubic (BCC) solid solution. Table 1 provides a detailed breakdown of the chemical composition of the powder, which confirms that the five principal alloying components are present in equal molar ratios. A comprehensive system that includes a laser device, a programmable robotic arm (KUKA), a cladding head, and a custom-built ultrasonic setup was employed to apply laser-clad coatings to 304 stainless steel, as depicted in Figure 2. The ultrasonic setup was specifically built to apply high-entropy alloy (HEA) coatings using resonant ultrasonic vibration-assisted laser cladding. The KUKA system utilizes a coaxial powder feeding nozzle that has a maximum power output of 4000 W. The ultrasonic setup works at a constant frequency of 20 kHz. A total of six coatings were created, each with different ultrasonic and laser settings, in order to investigate the impact of dilution on both microstructure and mechanical properties. The laser was configured to a power of (a) 1500 W, 5 µm, (b) 1500 W, 5, (c) 1700 W, 5 µm, (d) 1700 W, 10 µm, (e) 2100 W, 5 µm, and (f) 2100 W, 10 µm, with a consistent cladding rate of 7 mm/s and a flow rate of 7 L/min. The diameter of the laser spot utilized was 3 mm. Argon gas was utilized at a flow rate of 15 L/min for the experiment in order to prevent oxidation during the cladding process.

The equiatomic AlCrFeMnNi high-entropy alloy coating was applied to the AISI 304L steel using six different dilution rates, ranging from 30 to 60. The XRD study was conducted using CuKα radiation with a wavelength of 1.54 Å, using a Bruker D8 Advance Davinci instrument produced by (Billerica, MA, USA). The powders and coatings were analyzed using a scanning electron microscope (SEM, FEI Quanta 250 FEG), which was equipped with energy dispersive spectroscopy (EDS) capabilities. The cross-sectional microhardness of the coatings was measured using a Vickers indenter with a 100 g load and a dwell duration of 15 s. Friction testing was conducted in dry conditions using a GF-I multifunctional material surface performance tester at ambient temperature. The counterpart is composed of round Si_3_N_4_ material with a diameter of 4 mm. The applied force is 10 N, with a rotary speed of 280 r/min, sustained for a duration of 20 min. In addition, a TF20 TEM apparatus was used to conduct microstructure and phase analysis.

## 3. Results and Discussion

### 3.1. Dilution Rate

**Figure 3:**
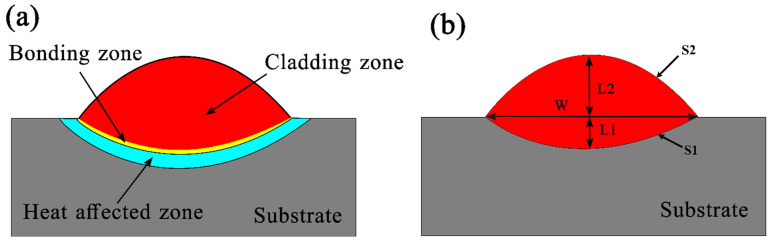
(**a**) Schematic illustration showing the different zones of a laser cladding, (**b**) showing the geometric factors of the laser cladding coating, CZ (coating zone), DZ (diffusion zone), *S*_1_ = area of the arc above the substrate, and (*S*_2_) area of the arc below the substrate. *L*_1_, height of the CZ; *L*_2_, height of the DZ; and *W*, the width of the coating.

The R-UVALC cladding process involves two distinct zones, each with specific characteristics and locations relative to the substrate surface. The first zone, termed the clad zone (CZ), is positioned above the substrate surface and corresponds to the *S*_2_ region, as illustrated in Figure 3a. This region is where the primary cladding action occurs. The second zone, known as the diffusion zone (DZ), is situated below the substrate surface, and its corresponding area is labeled as *S*_1_, as shown in Figure 3b. The DZ is where interactions between the cladding material and the substrate material take place, leading to diffusion-related phenomena. The dilution rate η, which quantifies the degree of mixing between the cladding material and the substrate material, can be calculated using Equation (1).

(1) η=S1S1+S2

In this specific context, *S*_1_ represents the area where the coating infiltrates the substrate, while *S*_2_ indicates the segment of the coating positioned above the substrate’s surface. The structural appearance of these coatings can be elucidated by categorizing them into two arcs, distinguished by their respective radii. Calculating the dimensions of *S*_1_ and *S*_2_ involves accounting for various geometric factors such as *L*_2_, which denotes the thickness of the clad layer, *L*_1_, which represents the thickness of the laser penetration layer, and W, which indicates the breadth of the coating.

(2) S1=W22+L122L12×arcsinW.L1L12+W22−WW22−L124L1

(3) S2=W22+L222L22×arcsinW.L2L22+W22−WW22−L224L2

**Figure 4:**
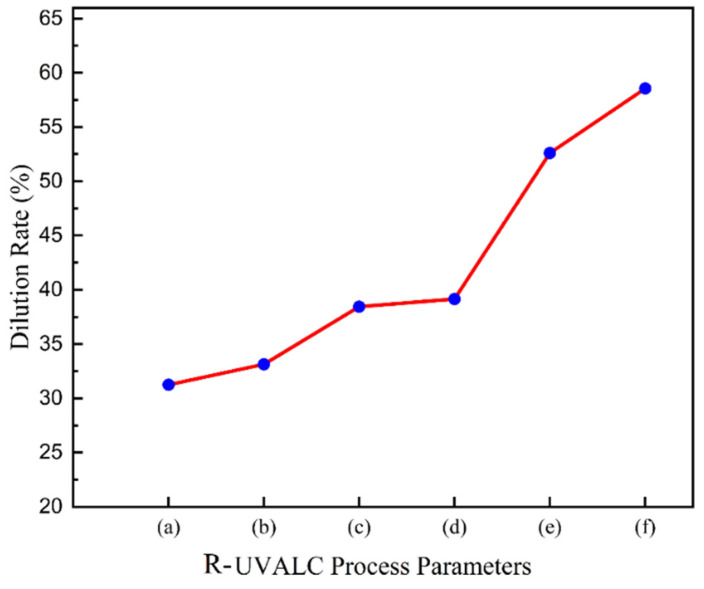
Dilution rates ((a) 31%, (b) 33%, (c) 37%, (d) 39%, (e) 53%, and (f) 60%) of the coating with different R-UVALC process parameters.

By using the above Equations (2) and (3), the dilute rate was calculated according to the profiles as 31%, 33%, 37%, 39%, 53%, and 60%, as shown in Figure 4, respectively.

**Figure 5:**
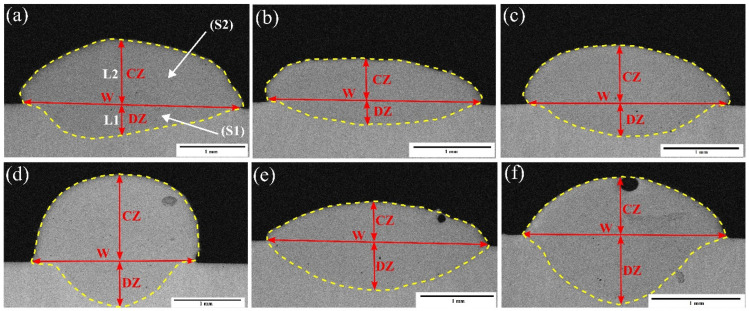
Cross-sectional morphology of the coating under six different dilution rates ((**a**) 31%, (**b**) 33%, (**c**) 37%, (**d**) 39%, (**e**) 53%, and (**f**) 60%) of the coating with different R-UVALC process parameters. CZ (coating zone), DZ (diffusion zone), *S*_1_ = area of the arc above the substrate, and (*S*_2_) area of the arc below the substrate. *L*_1_, height of the CZ; *L*_2_, height of the DZ; and *W*, the width of the coating.

Figure 5 exhibits the cladding profile of the examined samples, illustrating their appearance at six distinct dilution rates. This enables us to see and compare the different levels of dilution displayed by each sample. After doing a thorough examination of the microstructure, it is evident that the coatings display a dense and orderly arrangement, without any observable gaps or cracks. This demonstrates a significant degree of integrity and consistency in both the composition and deposition process of the coating. Furthermore, the coatings exhibit a robust metallurgical connection with the underlying material, indicating exceptional adherence and compatibility between the coating and substrate. As the dilution rates rise, there is a trend of increasing width in the coatings. The dimensions of the coatings, including their width and thickness, are directly linked to the amount of laser energy absorbed by the pre-deposited layers, which, in turn, is influenced by the amplitude of ultrasonic vibration in the R-UVALC setup. The width of the coating is directly proportional to both the amplitude of ultrasonic vibration and the processing parameters of laser cladding. Meanwhile, the thickness of the coating is governed by the total cross-sectional area and the width of the coating.

### 3.2. Phase Transformation

**Figure 6:**
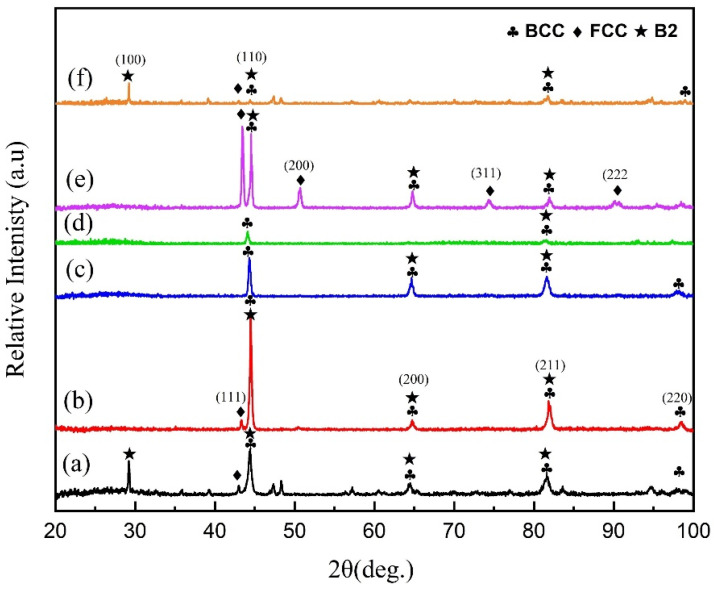
XRD pattern of the coating under different dilution rates ((**a**) 31%, (**b**) 33%, (**c**) 37%, (**d**) 39%, (**e**) 53%, and (**f**) 60%).

The XRD patterns presented in Figure 6 showcase the AlCrFeMnNi HEA coatings under various dilution rates. The analysis of phase characteristics reveals a significant correlation between the dilution rate and the phase composition of these coatings. Both an increase and decrease in the dilution rate trigger a phase transition in the AlCrFeMnNi HEA coatings, shifting from a BCC solid solution to a dual BCC and FCC solid solution. The valence electron concentration (VEC) of the Fe and Ni elements, along with the Cr, Co, Mn, and Al in the alloy, play a crucial role [18]. Fe and Ni have a relatively high VEC [8], impacting the overall VEC of the AlCrFeMnNi HEA coating. Fe increases in value as the dilution rate increases because of the Fe diffused into the coating from the substrate. Dual phases at a lower dilution rate can also be observed due to the higher amount of Ni content as the lower dilution rate results in a higher amount of Ni, which also helps in the generation of FCC due to a higher VEC value. On the other hand, at a higher dilution rate, this elevated VEC due to diluted Fe content contributes to the formation of the FCC phase [19]. Hence, when preparing HEA coatings via laser cladding, close attention should be paid to the dilution rate as it directly influences the phase structure and properties of the coatings. HEA coatings with low dilution rates often exhibit poor formability due to insufficient melting, while high dilution rates can significantly alter the phase structure, thereby impacting the properties of the coatings. Irrespective of high dilution results in dual phases, it can be observed that the lower and higher amounts of dilution both result in dual phases during the R-UVALC coating. Therefore, optimizing the dilution rate is crucial to achieving the desired phase composition and properties in HEA coatings prepared via laser cladding.

**Figure 7:**
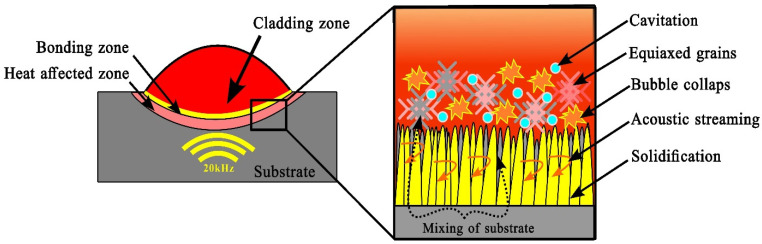
Schematic of how ultrasound induces acoustic cavitation and streaming in liquid melt pool of R-UVALC, vigorously stirring the melt during solidification.

**Figure 8:**
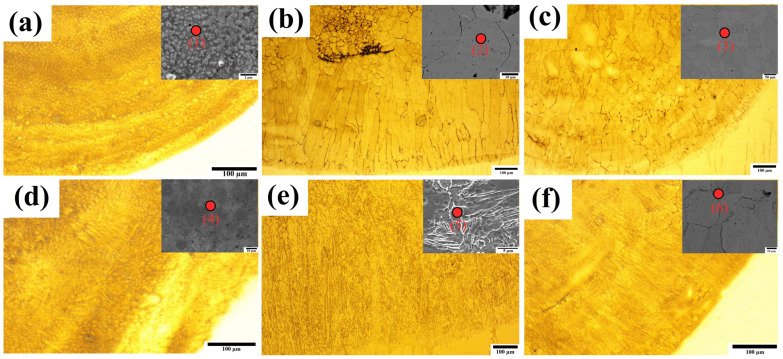
Microstructure of HEA R-UVALC coating under different dilution rates (**a**) 31, (**b**) 33, (**c**) 37, (**d**) 39, (**e**) 53, and (**f**) 60, with subset indicating magnified image with point EDS.

Figure 7 shows the schematic diagram of the bonding, cladding, and heat-affected zones during the R-UVALC. The grain growth in the high-entropy alloy (HEA) coatings was shown to proceed from the substrate toward the top of the coatings, mostly due to the substrate’s comparatively low temperature. The microstructural evolution of AlCrFeMnNi high-entropy alloy coatings with varying dilution rates was further investigated, and the results are presented in Figure 8a–f. Each dilution rate corresponds to a distinct microstructure profile, highlighting the profound influence of dilution rate on microstructural characteristics. The solidification process results in the creation of smaller grains with a grain structure that is equiaxed. In addition, the columnar grains are diminished at the interface with the substrate. The reason for this is attributed to the phenomena of acoustic streaming and acoustic cavitation. During the process of cavitation and bubble collapse, the substrate materials rise and blend with the coating, leading to an increase in the dilution of the coating. This, in turn, has a further impact on the microstructure and mechanical properties. Additionally, these effects improve the development of grain boundaries with tiny nuclei, which subsequently facilitates the initiation of nucleation and the creation of small grains [20,21].

At the lowest dilution rate, the microstructure exhibits an average grain size with visible precipitates within the grains, indicating the presence of the B2 phase. This observation is further supported by the XRD pattern in Figure 6, which clearly shows the characteristic line of the B2 phase. The microstructure profiles vividly depict this phase. As the dilution rate increases, a notable shift in grain structure occurs. Initially, elongated columnar grains emerge near the substrate, transitioning to equiaxed grains in the middle of the coating. With a further increase in dilution rate, a more uniform microstructure is achieved, characterized by fewer elongated columnar grains near the substrate and the prevalence of small equiaxed grains throughout the coating. Beyond a dilution rate of 37%, a non-uniform structure begins to emerge, accompanied by the appearance of an FCC peak and elongated columnar dendrites. It is noteworthy that the 37% dilution rate serves as a threshold for attaining a single-phase and uniform microstructure in the AlCrFeMnNi high-entropy alloy with 304L steel. Detailed analysis using SEM and EDS techniques revealed a correlation between the increasing dilution rate and the atomic percentage of Fe, as shown in Table 2. As the dilution rate rises, there is a corresponding increase in the atomic percentage of Fe, further emphasizing the intricate relationship between dilution rate, microstructural evolution, and elemental composition in high-entropy alloy coatings.

As the dilution rate increases, different phase transformations can be observed. The lowest and greatest dilution values lead to an uneven microstructure with two phases present. However, with a dilution rate of 37%, a single phase of BCC is formed with B2 precipitates inside. The phase characterization outcomes indicate that the dilution rate has a substantial impact on the phase makeup of AlCrFeMnNi high-entropy alloy coatings. An increase in the dilution rate results in a phase change in the AlCrFeMnNi HEA coatings, transitioning from a body-centered cubic (BCC) solid solution to a face-centered cubic (FCC) dual solid solution. The EDS analysis of points (1–6) shows that the iron (Fe) content increases with the dilution rate. Among the elements in the alloy, iron (Fe with a valence electron concentration, VEC, of 8), chromium (Cr with a VEC of 6), cobalt (Co with a VEC of 9), manganese (Mn with a VEC of 7), and aluminum (Al with a VEC of 3), iron has a relatively high VEC. This means that as the Fe content is diluted, the VEC of the AlCrFeMnNi high-entropy alloy (HEA) coating increases, which, in turn, promotes the formation of the face-centered cubic (FCC) phase [22,23,24].

### 3.3. Microhardness

**Figure 9:**
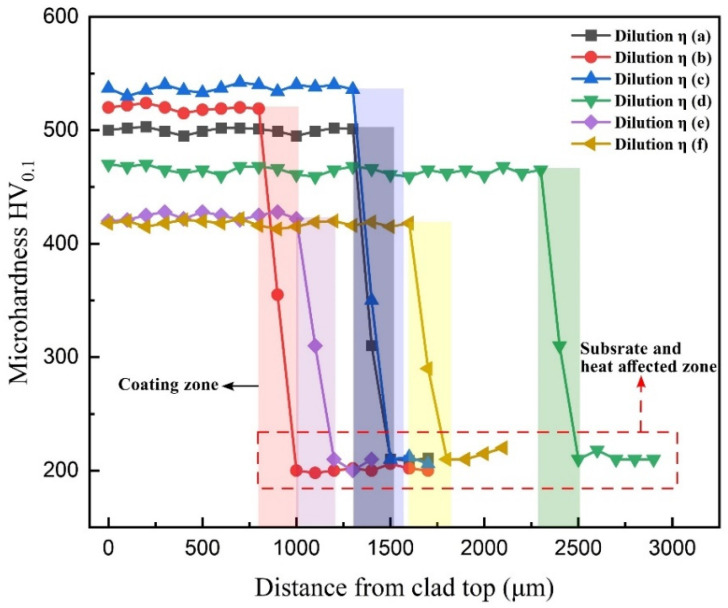
Microhardness profile of the HEA R-UVALC coating under different dilution rates.

The microhardness profile, as depicted in Figure 9, reveals intriguing insights into the effect of dilution and phase composition on material properties. It is evident that dilution leading to the presence of dual phases correlates with a lower microhardness profile. Conversely, the attainment of a single-phase BCC structure at a dilution rate of 37% yields the highest microhardness values. This observation aligns with established criteria for AlCrFeMnNi high-entropy alloy (HEA) coatings, where the control of the FCC/BCC phase ratio plays a pivotal role in achieving exceptional combinations of strength and ductility. It is well-documented that alloys comprising both FCC and BCC phases can exhibit unique mechanical properties. In general, the hardness of the FCC phase falls within the range of 1.0 to 2.0 GPa [25]. Furthermore, HEAs predominantly composed of the FCC phase, such as CoCrCuFeNi, demonstrate ductilities ranging from 20% to 60% and often exhibit substantial work-hardening [26,27]. The highest amount of microhardness was achieved with the dilution of 37%, which is a single-phase BCC solid solution with B2 precipitates inside it. Deviating from this dilution rate results in a lower microhardness profile.

**Figure 10:**
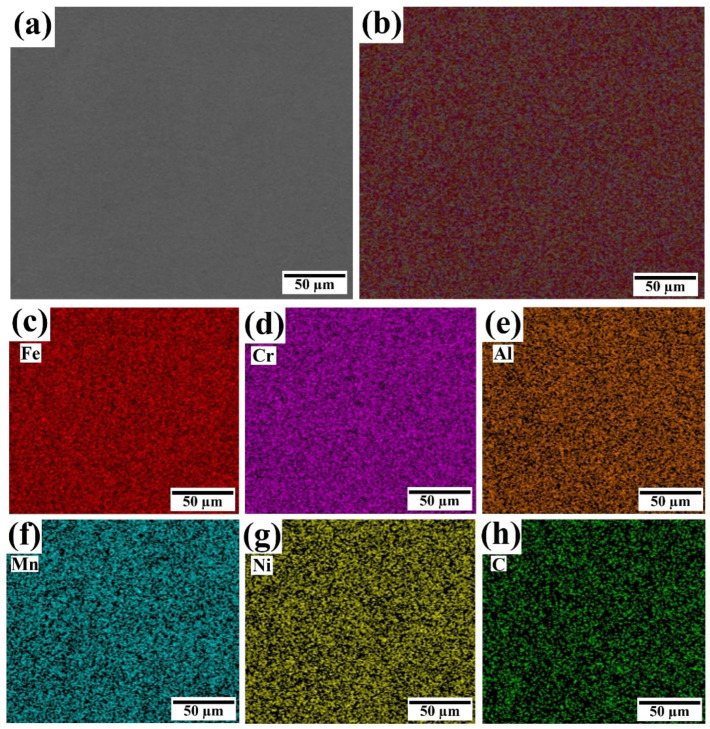
EDS mapping of the coating at the optimum value of dilution rate of 37% (**a**) selected portion of the R-UVALC coating surface, (**b**) element distribution mapping of the selected portion of the coating, and (**c**–**h**) element distribution mapping of each element Fe, Cr, Al, Mn, Ni, and C, respectively.

To delve deeper into the microhardness observations at the 37% dilution rate, an EDS mapping analysis was conducted to visualize the distribution of elements within the coating, as shown in Figure 10. This analysis revealed a homogeneous distribution of all elements, contributing to the uniformity and enhanced mechanical properties observed in the microhardness profile. These findings underscore the importance of phase control and elemental distribution in tailoring the mechanical behavior of HEA coatings, particularly in achieving superior microhardness characteristics.

### 3.4. Friction Coefficient

**Figure 11:**
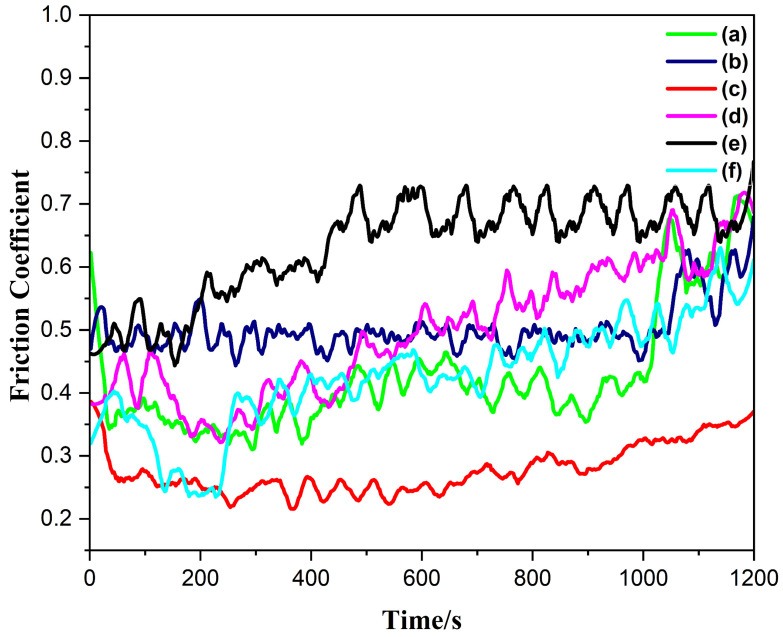
Friction coefficient values of ultrasonic vibration-assisted coating of HEAs at ambient temperature with varying dilution rates (a–f).

The friction coefficient is a crucial parameter for assessing the tribological properties of laser cladding layers. The friction coefficient was evaluated at room temperature over six dilution rates: the minimal friction coefficient was attained at (c), which corresponds to the second lowest dilution rate, whereas elevated friction coefficient values were noted as the dilution rate increased. A consistent coefficient of friction was additionally measured at (c) with the lowest dilution rate, as seen in Figure 11. The minimal friction coefficient values at (c) rates indicate that the coatings exhibit exceptional antifriction properties. The smallest coefficient of friction at this dilution rate is also consistent with the maximum microhardness. The elevation in the friction coefficient at elevated dilution rates can be ascribed to the formation of both face-centered cubic (FCC) and body-centered cubic (BCC) phases at increased amplitudes.

### 3.5. TEM Analysis

**Figure 12:**
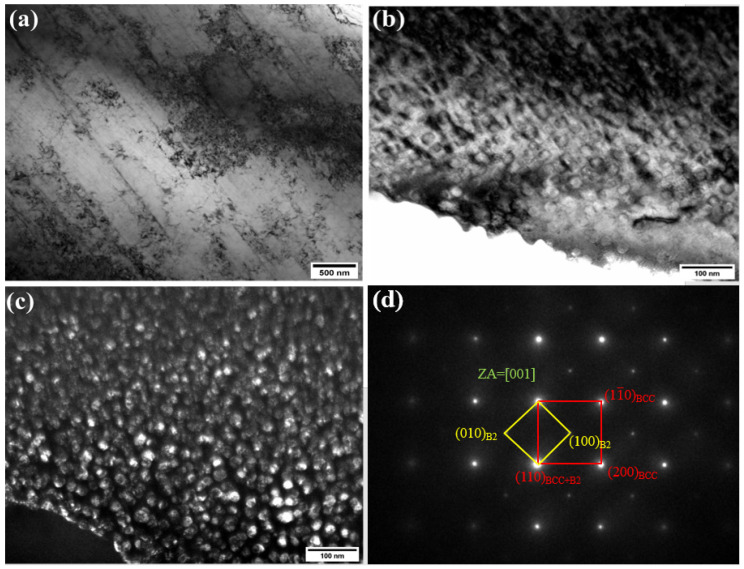
TEM of the coating at the optimum dilution rate of 37%. (**a**) TEM micrograph, (**b**) bright field image, (**c**) dark field image of the coating, showing the cuboidal B2 precipitates, and (**d**) SAED pattern of the R-UVALC, showing the presence of B2 precipitates.

The presence of B2 precipitates results in the highest value of microhardness, which further increases the wear-resistance properties of the laser-clad coating. To visualize the presence of B2 precipitates inside the BCC phase, TEM analysis was performed on the optimum dilution sample of 37%. Additionally, cuboidal precipitates were identified within the body-centered cubic (BCC) phase of the clad coating. Analysis of selected area electron diffraction (SAED) patterns indicated that these precipitates exhibit a B2 crystal structure. A chemical composition analysis of the cuboidal precipitates within the alloy showed that they are primarily enriched with aluminum (Al) and nickel (Ni). Figure 12a shows the image of the coating at 37% dilution rate. Figure 12b shows the dark field image, showing the presence of cuboidal precipitates that are further inducted in the bright field image. The SAED pattern in Figure 12c reveals the presence of B2 precipitates inside the main BCC phase.

## 4. Conclusions

The research findings underscore the significant influence of dilution in ultrasonic vibration-assisted laser cladding on microstructural evolution and phase transformation. An increase in the dilution rate correlates with higher Fe content, impacting the resulting microstructure. Notably, both high and low dilution rates lead to the formation of dual phases within the coating. A 37% dilution rate results in the lowest friction coefficient, which results in the highest antifrictional properties.

The presence of dual phases, particularly at extreme dilution rates, contributes to distinct changes in the microstructure profile, consequently affecting the microhardness properties of the coating. Through transmission electron microscopy (TEM) analysis, B2 precipitates were identified within the BCC phase, and were primarily composed of Al and Ni elements. These B2 precipitates significantly contribute to the enhancement of the microhardness profile and are anticipated to improve the wear and tribological properties of the coating.

The role of dilution in ultrasonic vibration-assisted laser cladding cannot be overstated, as it not only influences the phase composition but also impacts the mechanical and tribological properties, particularly microhardness and antifriction properties. These findings provide valuable insights for optimizing processing parameters and designing advanced coatings with superior performance characteristics. Furthermore, R-UVALC possesses significant potential for laser cladding of challenging clad materials, including titanium and aluminum alloys.

## Figures and Tables

**Table 1:** The chemical composition of the AlCrFeMnNi high-entropy alloy.

Elements	Al	Cr	Fe	Mn	Ni
At%	20.89	19.25	18.02	22.26	19.58
Wt%	11.40	20.25	20.35	24.74	23.26

**Table 2:** Point EDS of the laser-clad coating.

Elements (at. %)	Fe	Al	Ni	Mn	Cr	C
Point 1	35.40	5.66	14.42	7.96	18.77	17.79
Point 2	37.42	5.35	14.46	7.31	17.91	17.55
Point 3	38.38	8.49	14.44	5.76	14.61	18.32
Point 4	45.33	1.96	12.34	5.33	12.41	22.63
Point 5	48.15	3.45	11.91	4.24	11.11	21.14
Point 6	50.23	6.80	10.53	2.32	10.02	20.10

## Data Availability

The original contributions presented in this study are included in the article. Further inquiries can be directed to the corresponding authors.

## References

[1] Yeh J.-W., Chen S.K., Lin S.-J., Gan J.-Y., Chin T.-S., Shun T.-T., Tsau C.-H., Chang S.-Y. (2004). Nanostructured high-entropy alloys with multiple principal elements: Novel alloy design concepts and outcomes. Adv. Eng. Mater..

[2] Cantor B., Chang I.T.H., Knight P., Vincent A.J.B. (2004). Microstructural development in equiatomic multicomponent alloys. Mater. Sci. Eng. A.

[3] Jiang H., Han K., Li D., Cao Z. (2019). Synthesis and characterization of AlCoCrFeNiNb_x_ high-entropy alloy coatings by laser cladding. Crystals.

[4] Liu S., Zhang M., Zhao G., Wang X., Wang J. (2022). Microstructure and properties of ceramic particle reinforced FeCoNiCrMnTi high entropy alloy laser cladding coating. Intermetallics.

[5] Wang W.R., Wang W.L., Wang S.C., Tsai Y.C., Lai C.H., Yeh J.W. (2012). Effects of Al addition on the microstructure and mechanical property of AlxCoCrFeNi high-entropy alloys. Intermetallics.

[6] Yang Y., Yang S., Wang H. (2021). Effects of the phase content on dynamic damage evolution in Fe50Mn30Co10Cr10 high entropy alloy. J. Alloys Compd..

[7] Wu H., Zhang S., Wang Z.Y., Zhang C.H., Zhang D.X., Chen H.T., Wu C.L. (2022). Phase evolution, microstructure, microhardness and corrosion performance of CoCrFeNiNb x high entropy alloy coatings on 316 stainless steel fabricated by laser cladding. Corros. Eng. Sci. Technol..

[8] Wu H., Zhang S., Wang Z., Zhang C., Chen H., Chen J. (2022). New studies on wear and corrosion behavior of laser cladding FeNiCoCrMox high entropy alloy coating: The role of Mo. Int. J. Refract. Met. Hard Mater..

[9] Munitz A., Meshi L., Kaufman M. (2017). Heat treatments’ effects on the microstructure and mechanical properties of an equiatomic Al-Cr-Fe-Mn-Ni high entropy alloy. Mater. Sci. Eng. A.

[10] Mohsan A.U.H., Zhang M., Wang D., Zhao S., Wang Y., Chen C., Zhang J. (2023). State-of-the-art review on the Ultrasonic Vibration Assisted Laser Cladding (UVALC). J. Manuf. Process..

[11] Shon Y., Joshi S.S., Katakam S., Rajamure R.S., Dahotre N.B. (2015). Laser additive synthesis of high entropy alloy coating on aluminum: Corrosion behavior. Mater. Lett..

[12] Mohsan A.U.H., Zhang M., Wang D., Wang Y., Zhang J., Zhou Y., Li Y., Zhao S. (2024). Design and Effect of Resonant Ultrasonic Vibration-Assisted Laser Cladding (R-UVALC) on AlCrFeMnNi High-Entropy Alloy. Materials.

[13] Zhu L., Yang Z., Xin B., Wang S., Meng G., Ning J., Xue P. (2021). Microstructure and mechanical properties of parts formed by ultrasonic vibration-assisted laser cladding of Inconel 718. Surf. Coat. Technol..

[14] Zhang M., Zhao G., Wang X., Liu S., Ying W. (2020). Microstructure evolution and properties of in-situ ceramic particles reinforced Fe-based composite coating produced by ultrasonic vibration assisted laser cladding processing. Surf. Coatings Technol..

[15] Kim J.-D., Peng Y. (2000). Melt pool shape and dilution of laser cladding with wire feeding. J. Mater. Process. Technol..

[16] Xu P., Li P., Chen Y., Wang B., Lu H., Xu C., Dai M. (2024). Optimization of process parameters for laser cladding Stellite6 cobalt-based alloy. Mater. Today Commun..

[17] Hemmati I., Ocelík V., De Hosson J.T.M. (2012). Dilution effects in laser cladding of Ni–Cr–B–Si–C hardfacing alloys. Mater. Lett..

[18] Cui C., Wu M., Miao X., Zhao Z., Gong Y. (2022). Microstructure and corrosion behavior of CeO2/FeCoNiCrMo high-entropy alloy coating prepared by laser cladding. J. Alloys Compd..

[19] Liu L., Zhang Y., Han J., Wang X., Jiang W., Liu C.T., Zhang Z., Liaw P.K. (2021). Nanoprecipitate-strengthened high-entropy alloys. Adv. Sci..

[20] Fan Q., Chen C., Fan C., Liu Z., Cai X., Lin S., Yang C. (2021). Ultrasonic induces grain refinement in gas tungsten arc cladding AlCoCrFeNi high-entropy alloy coatings. Mater. Sci. Eng. A.

[21] Ma G., Yan S., Wu D., Miao Q., Liu M., Niu F. (2017). Microstructure evolution and mechanical properties of ultrasonic assisted laser clad yttria stabilized zirconia coating. Ceram. Int..

[22] Chen Q., Zhou K., Jiang L., Lu Y., Li T. (2015). Effects of Fe content on microstructures and properties of AlCoCrFe x Ni high-entropy alloys. Arab. J. Sci. Eng..

[23] Yin K., Dong G., Zhang G., Tian Q., Wang Y., Huang J. (2023). Prediction of phase structures of solid solutions for high entropy alloys. J. Mater. Res. Technol..

[24] Yang S., Lu J., Xing F., Zhang L., Zhong Y. (2020). Revisit the VEC rule in high entropy alloys (HEAs) with high-throughput CALPHAD approach and its applications for material design-A case study with Al–Co–Cr–Fe–Ni system. Acta Mater..

[25] Mishra R.S., Haridas R.S., Agrawal P. (2021). High entropy alloys—Tunability of deformation mechanisms through integration of compositional and microstructural domains. Mater. Sci. Eng. A.

[26] Hassan M.A., Ghayad I., Mohamed A., El-Nikhaily A.E., Elkady O.A. (2021). Improvement ductility and corrosion resistance of CoCrFeNi and AlCoCrFeNi HEAs by electroless copper technique. J. Mater. Res. Technol..

[27] Ng C., Guo S., Luan J., Wang Q., Lu J., Shi S., Liu C. (2014). Phase stability and tensile properties of Co-free Al0.5CrCuFeNi2 high-entropy alloys. J. Alloys Compd..

